# Functional Abstraction as a Method to Discover Knowledge in Gene Ontologies

**DOI:** 10.1371/journal.pone.0090191

**Published:** 2014-02-25

**Authors:** Alfred Ultsch, Jörn Lötsch

**Affiliations:** 1 DataBionics Research Group, University of Marburg, Marburg, Germany; 2 Institute of Clinical Pharmacology, Goethe - University, Frankfurt am Main, Germany; 3 Fraunhofer Institute for Molecular Biology and Applied Ecology IME, Project Group Translational Medicine and Pharmacology TMP, Frankfurt am Main, Germany; Nazarbayev University, Kazakhstan

## Abstract

Computational analyses of functions of gene sets obtained in microarray analyses or by topical database searches are increasingly important in biology. To understand their functions, the sets are usually mapped to Gene Ontology knowledge bases by means of over-representation analysis (ORA). Its result represents the specific knowledge of the functionality of the gene set. However, the specific ontology typically consists of many terms and relationships, hindering the understanding of the ‘main story’. We developed a methodology to identify a comprehensibly small number of GO terms as “headlines” of the specific ontology allowing to understand all central aspects of the roles of the involved genes. The Functional Abstraction method finds a set of headlines that is specific enough to cover all details of a specific ontology and is abstract enough for human comprehension. This method exceeds the classical approaches at ORA abstraction and by focusing on information rather than decorrelation of GO terms, it directly targets human comprehension. Functional abstraction provides, with a maximum of certainty, information value, coverage and conciseness, a representation of the biological functions in a gene set plays a role. This is the necessary means to interpret complex Gene Ontology results thus strengthening the role of functional genomics in biomarker and drug discovery.

## Introduction

The computational analysis of complex biological pathways has become an increasingly important part of biology. To reveal interaction networks of complex traits and diseases from sets of genes obtained from microarray analyses, proteomic research or thematic literature searches, the knowledge captured in cell biological ontologies is exploited. The gold-standard in this field is the Gene Ontology (GO; http://www.geneontology.org/) [1] where the major biological processes, cellular components or molecular functions of the genes respectively gene products are described by a controlled vocabulary (GO terms) [2]. A characterization of a gene set is obtained by statistical means identifying GO terms that are overrepresented in the gene list, i.e., annotated to the gene list more often than expected by chance [3], [4].

**Figure 1:**
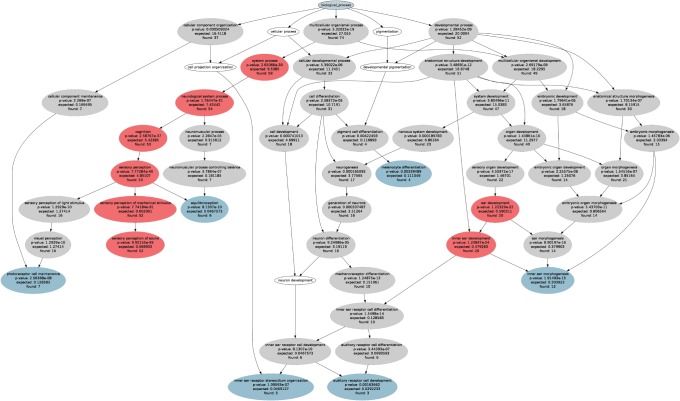
ORA results and functional areas obtained with the CLASSIC abstraction methods. Graphical representation of the specific ontology showing the polyhierarchy of functional annotations (GO terms) assigned to HHI gene set (G = 119, Table 1) and forming a directed acyclic graph (DAG). The figure was generated with the GeneTrail web-based analysis tool [12]. Significant GO terms were identified using ORA, which resulted in 71 terms at a significance level of p = 1.0 · 10^−2^ and Bonferroni α correction (grey ellipses in which the observed number of member genes, the expected number of genes by chance and the p-value of the significance of the deviation from the expectations (Fisher’s exact test) are annotated). The CLASSIC p-value approach to the interpretation of ORA results is the selection of headline terms along descending statistical significance. When setting the p-value threshold at p = 10^−20^, eight headlines resulted (red ellipses). The CLASSIC detail approach is the selection of the leaves of each ontology, which with the present ORA parameters resulted in seven details (blue ellipses plus “sensory perception of sound, the latter colored red since also selected by the p-value method).

However, the intended comprehension of main processes and interaction networks characterizing a gene set is often impeded by the complexity of the results of such an over-representation analysis (ORA) (Figure 1). A complete representation of the knowledge about the gene set’s function as result of an ORA is contained in a specific ontology, which is a directed acyclic graph (DAG, knowledge representation graph). Such a specific ontology often contains hundreds of significant terms and therefore fails to provide a comprehensible selection of relevant information on the functionality of the given gene set. Therefore, an abstraction method is needed. Classical approaches, i.e., choosing most significant or most specialized terms, provided only narrowed views on the functions represented in a gene set. Other approaches were focused on the decorrelation of GO terms [5].

The proposed methodology of functional abstraction aims at identifying a small number of GO terms (headlines) that confer the “big picture” of the biological functions of the genes in a set of genes. Its main goal was providing an informative representation that covers the different aspects of biological functions of a gene set at a human-understandable level [6].

## Methods

### Selection of Gene Sets

To demonstrate this knowledge discovery method on a real-life example, a set of genes which are known to be associated with a specific research topic is selected. Such a topical set of genes causally associated with hearing impairment [7] was retrieved mainly (n = 104 genes) from the “Hereditary Hearing Loss Homepage” at http://hereditaryhearingloss.org on September 20, 2013. The causal genotype phenotype associations in that data base correspond to the recommendations of the GENDEAF study group at http://hereditaryhearingloss.org/main.aspx?c=.HHH&n=86638. Additional genes (n = 6) were obtained from [8] and from the Deafness Gene Mutation Database at http://hearing.harvard.edu/db/genelist.htm, and further genes (n = 9) were added from a recently actualized review [9]. The complete set of *n* = 119 genes (Table 1) is referred to as the Hereditary Hearing Impairment (HHI) gene set intended as a didactical example with therefore few genes in comparison to previous methodologically similar analyses (e.g., 410 genes in a topical set of pain genes [10], 231 genes in the microarray derived expression pattern of the olfactory bulb [11]).

**Table 1:** The hereditary hearing impairment (HHI) example data set, consisting of G = 119 genes (for names and functional explanations, see http://www.genenames.org/or Table S1, HearingGenes119.xlsx) taken mainly from the Hereditary Hearing Loss Homepage at http://hereditaryhearingloss.org [7] on September 20, 2013 (104 genes) and completed from the Deafness Gene Mutation Database at http://hearing.harvard.edu/db/genelist.htm, and two publications, i.e., [8] and [9] with its last revision dating from January 3, 2013 (http://www.ncbi.nlm.nih.gov/books/NBK1434/).

*ACTG1*	*CLRN1*	*DFNB31*	*FAM189A2*	*HARS2*	*LRTOMT*	*MYO15A*	*PCDH15*	*SLC17A8*	*TMC1*
AGAP2	COCH	DFNB59	FOXI1	HGF	MARVELD2	MYO1A	PDZD7	SLC26A4	TMIE
ATP2B2	COL11A1	DIABLO	GIPC3	HSD17B4	MIR96	MYO1C	POU3F4	SLC26A5	TMPRSS3
BSND	COL11A2	DIAPH1	GJA1	ILDR1	MITF	MYO1F	POU4F3	SMPX	TMPRSS5
CABP2	COL2A1	DIAPH3	GJB1	KCNE1	MSRB3	MYO3A	PRPS1	SNAI2	TPRN
CCDC50	COL4A3	DSPP	GJB2	KCNJ10	MT-RNR1	MYO6	PTGS1	SOX10	TRIOBP
CDH23	COL4A4	EDN3	GJB3	KCNQ1	MT-TE	MYO7A	PTPRQ	SOX2	TRMU
CEACAM16	COL4A5	EDNRB	GJB6	KCNQ4	MT-TK	NDP	RDX	STRC	USH1C
CHD7	COL9A1	ESPN	GPR98	KIAA1199	MT-TL1	NF2	SEMA3E	TCOF1	USH1G
CIB2	COL9A2	ESRRB	GPSM2	LARS2	MT-TS1	OTOA	SERPINB6	TECTA	USH2A
CLDN14	CRYM	EYA1	GRHL2	LHFPL5	MYH14	OTOF	SIX1	TIMM8A	WFS1
CLPP	DFNA5	EYA4	GRXCR1	LOXHD1	MYH9	PAX3	SIX5	TJP2	

### Gene Over-representation Analysis (ORA)

Subcategories of biological functions in which the genes of the example set are involved were identified by means of ORA [3] using the web-based GeneTrail [12] tool at http://genetrail.bioinf.uni-sb.de/. This compared the GO terms annotated to the expressed genes with the occurrence of terms among the set of all human genes. The significance of a GO term associated with the present list of genes was determined by means of a hypergeometric test that annotates the resulting GO terms with p-values. Subsequently, a correction for multiple testing was applied and only terms with a p-value lower than preset threshold *t_p_* were considered as significant. For the HHI gene set, the threshold *t_p_*, was set at 0.01 (similarly as elsewhere used [5]) and corrected for multiple testing according to Bonferroni, which resulted in a *significant term set* (for definitions, see Table 2).

**Table 2:** Definitions and notations used in the present functional abstraction process.

**Gene set:** a number of genes for which the genetic functionality is sought, often the result of other experiments such as microarray or proteomic analysis or database research for a certain topic such as “pain”.
**Overrepresentation analysis (ORA):** calculation of a significant term set for a gene set. For all terms *T_i_* of the GO p-values pval(*T_i_*) are calculated with regard to the gene annotations of *T_i_* and the gene set by using Fisher’s exact test statistic [3]. To obtain a significant term set usually a predefined threshold t_p_ is used and only terms *T_i_* with (pval(*T_i_*)<t_p_) are regarded, and corrections to control for multiple testing errors (e.g. Bonferroni, False Discovery Rate [19]) are applied.
**Significant term set:** the result of an ORA. The set of GO terms consisting of those terms that are annotated to the given gene set significantly more often than expected by chance. The significant terms set forms a specific ontology.
**Specific Ontology:** a subset of the GO, the polyhierarchy formed by the significant terms set within the thematic ontologies biological process, molecular function or cellular component.
**Root term/top level term:** the most general GO term of the thematic ontology from which all specific ontologies originate.
**Details:** the leaves of an ontology, describing the most specific pieces of knowledge.
**Taxonomy:** a path from the root term to a particular detail, narrowing the definitions of the terms from universal to specific details.
**Remarkableness of a term:** a non-negative number proportional to certainty and information value of a term.
**Headline:** the term with the largest remarkableness of taxonomy.
**Subsumption:** substitution of a set of headlines H = {T_1_,..T_k_} by a single term T. T must cover H i.e. all paths from the root term to any term in H must pass through T.
**Detailization:** substitution of a headline T by a set of terms H = {T_1_,..T_k_} which are covered by T.
**Functional Areas:** a set of terms FA = {T_1_,…,T_n_} covering all details of a specific ontology. FA optimizes certainty (P-values) and is most informative in an information theoretical sense. The size of the set is optimized such that human understanding is enhanced.

ORA provided a representation of what is known (knowledge representation) about the roles of the genes in an organism. The significant term set may derive specific ontologies starting from either of the three possible root terms, i.e., “biological process”, “cellular component” or “molecular function” [1]. In each specific ontology the terms are arranged in a polyhierarchy starting at the root, with the broadest definition, and specializing toward the leaves, with the narrowest definition (details). For the present analysis, “biological process” was chosen (Figure 1). This consists of one or more ordered assemblies of molecular functions involving chemical or physical transformations, such as cell growth and maintenance or signal transduction [1].

The resulting specific ontology contained 48 significant terms, with the most detailed descriptions of the role of HHI gene set specified in seven leaves (Figure 1). A path from the root term to a particular detail, narrowing the definitions of the terms from universal to specific, is called “taxonomy” (Figure 1). For example the path “biological process”, “multicellular organismal process”, “system process”, “neurological system process”, “cognition”, “sensory perception”, “equilibrioception” is the taxonomy for the detail “equilibrioception”.

### Abstraction of ORA Results

With typical sets of several hundred genes the resulting specific ontologies typically also contain 100 and more significant terms [10], [11]. Even for the present small HHI gene set of 119 genes and using restrictive multiple testing correction, the specific ontology contained approximately 50 significant terms. Identifying a manageable amount of terms as “**headlines**” of the “main story” will in the following be referred to as an **abstraction** of the specific ontology.

As quality criteria for understandable and informative subsets (abstractions) of significant GO terms (headlines) of a specific ontology, four dimensions were predefined, i.e., certainty, coverage, information value and conciseness.

*Firstly*, **certainty** requires that terms should be relevant for the gene set. For a term *T_i_* in the significant term set, the certainty measure was defined as *Cert(T_i_) = p(there is a Term with smaller P-Value) = (#(T_k_ with p-value<pval(T_i_)))/n_T_*, where *n_T_* denotes the number of significant GO terms annotated to the given set of genes. This reflects how safe it is to assume that the term *T_i_* describes the gene set, with numerical values in the interval [0,1]. The certainty of the whole abstraction is the average certainty of all headlines in this abstraction.

*Secondly*, **coverage** requires the headlines to incorporate all the details of a specific ontology in the abstraction. A term *T,* which is not the root, covers a term *T_d_*, if there is a path (in the direction from root to leaf) in the specific ontology from *T* to *T_d_*. The coverage of an abstraction can be measured as the percentage of covered details in the ontology.

**Figure 2:**
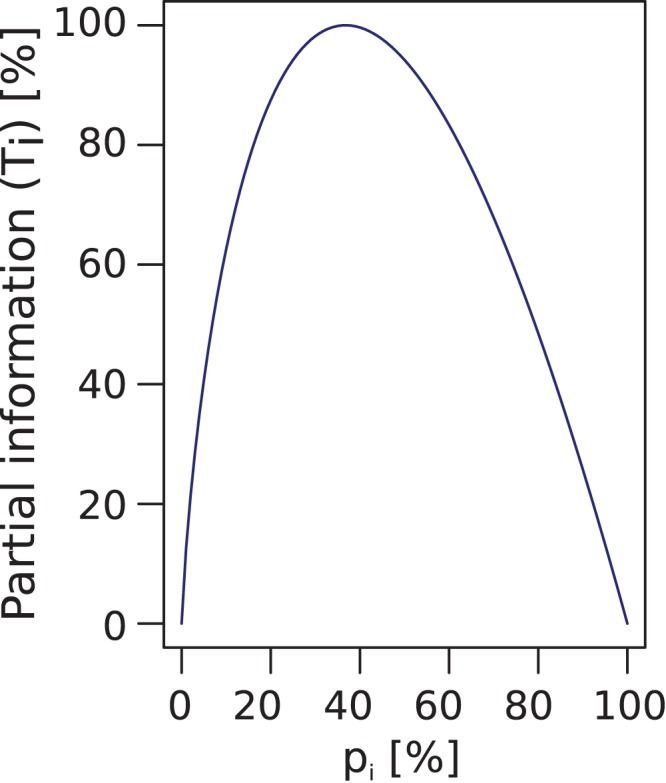
Graph of the Information value function *Info(T_i_) = −e • p_i_ • ln(p_i_)*, *p_i_ = n_G(Ti)_/n_G_*, where *n_G(Ti)_* denotes the number of genes of a set annotated to a term *T_i_* and *n_G_* denotes the total number of genes in the set. Derived from Shannon information [14], *Info(T_i_)* measures the contribution of the annotations of T_i_ to the total (Shannon) information of an specific ontology. Specifically, In bioinformatics, *IC(T_i_) = −log(p_i_)* measures the information content (IC) of a GO term, [21], if *p_i_* is the number of all genes annotated to *T_i_* relative to all annotations in the GO. So *Info(T_i_)* can be interpreted as weighted Information Content of a specific ontology. *Info(T_i_)* = 0 if term *T_i_* does not possess any annotations (*p_i_* = 0) and for the root of the ontology. *Info(T_i_)* has its maximum *Info(T_i_)* = 1 at a gene probability of 37%.

*Thirdly*, the **information** value requires that the identified headlines should be as informative as possible. To capture this dimension, the (partial) Shannon information of a term *T_i_* in the significant term set was calculated. For each *T_i,_* its gene frequency (probability) can be calculated: *p_i_ = n_G(Ti)_/n_G_*, where *n_G(Ti)_* denotes the number of genes of a set annotated to a term *T_i_* and *n_G_* denotes the total number of genes in the set. In information theory the (Shannon-) information or entropy of a probability distribution P = {p_1_,…, p_n_} is measured as [13], [14]. The terms in the summation, i.e. *Info(T_i_)*, measure the particular information (*information value*) that is contributed to the total entropy by the annotations of the particular term. Using the factor c = *e* and the natural logarithm, i.e. *Info(T_i_) = −e • p_i_ • ln(p_i_)*, scales the values of *Info(T_i_)* to the interval [0,1]. The graph of this function is arc shaped (Figure 2) reflecting that maximum information value is provided neither by the root term of the specific ontology, e.g., “biological process” which is relevant for all genes and due to its low information cannot be selected, nor by the details which describe the role of only a small number of genes, such as “potassium transport” may be too detailed when “sodium transport” is also important and therefore “ion transport” should be preferred.

*Fourthly*, the dimension of **conciseness** aims at a number of headlines facilitating that humans can grasp the specific ontology, as few as possible, however not too few to avoid very abstract but general headlines covering all details. A suitable approach to this requirement is the Miller number [15] of seven headlines. If there are less than 7 - 5 headlines some terms should be replaced by more detailed terms. If there are more than 7–9 headlines some terms should be merged. Ideally a number of 5–9 terms enhances human comprehension [16].

#### Classical approaches at ORA abstraction

The current state-of-the-art approaches to a concise interpretation of ORA results mainly consist of (i) selections of the most significant terms as headlines (CLASSIC p-value), (ii) detail method taking the leaves of the ontology (CLASSIC detail), or (iii) ad hoc selection. Considering the shortcomings of current approaches, it becomes evident that a new method providing a comprehensive coverage of the functions of a gene set is needed.

The selection of the terms with the smallest p-values as headlines is a classical approach for the selection of a meaningful subset of headline terms (CLASSIC p-value). For example, a p-value limit of less than 10^−20^ selects the eight headline terms marked in red in Figure 1. One essential requirement of headline selection is complete coverage of details. In a specific ontology this means that the taxonomies of all details are covered. At least one of the headlines (other than the root) should be on the path from the detail to the root. Using the CLASSIC p-value method, however, there are several details which are not covered by these headlines.

However, this covered the ontology only poorly since more than half of the details lacked a headline (Figure 1). A possible workaround would be taking all the details as headlines (CLASSIC detail). However, this included several uninformative headlines such as “photoreceptor cell maintenance” and “melanocyte differentiation”. This failed to provide an adequate overview about biologic functions concerned with hearing loss. Moreover, the results of these procedures critically depend on the parameters of the particular ORA. Therefore a different set of headlines would result from choosing other ORA significance levels. Sometimes the specific ontology is just eyeballed and a set of headline terms is ad hoc selected as particularly interesting. An example of such an approach can be found in [8]. There, for a gene set of 51 non-syndromic hereditary hearing loss genes, which is a subset of the present HHI, five headlines were identified in a specific ontology consisting of 42 terms (green circles in figure 4 of [8]). Four of these headlines are the details of the specific ontology, one is an arbitrarily chosen inner node.

#### Functional abstraction

To better meet the requirements at an abstraction than classical approaches and to obtain an understandable and informative set of GO terms from ORA, the following heuristic of functional abstraction (FA) was developed:

For each term *T_i_* in the set of terms, its remarkableness, *Rem(T_i_)*, was calculated as the product of **certainty** and **information value**, i.e., *Rem(T_i_) = Cert(T_i_)·Info(T_i_)*. **Coverage** was addressed by assuring that the taxonomies of all details of a specific ontology, i.e., all the different paths from the leaves (details) to the root, are being considered. Specifically, the most remarkable term in each taxonomy was headline candidate. From all candidate terms, C, redundancies were eliminated, i.e., if all parents of a term T in C were also members of C, then T was deleted as already represented in C, thereby addressing conciseness of the **abstraction**. The remaining headlines, *H*, of this FA are called “functional areas”.

While these functional areas are a suitable comprehensive representation of the taxonomies of a specific ontology, a more global abstraction can be obtained by two methods: detailization or subsumption. Let *T* be a term in a specific ontology which covers the terms *T_1_,..T_k_*. A set of headlines *H* containing *T* is detailed if *T* is replaced by the headlines *T_1_,..T_k_* in H. Alternatively, a set of headlines *H* containing *T_1_,..T_k_* is abstracted if *T_1_,..T_k_* are replaced by *T* in *H*. Detailization enlarges, subsumption reduces the number of headlines. Note that the root is never a headline, since it is excluded from the definition of coverage. To enhance human comprehensibility a number of 5–9 headlines correspond to the human capacity of information processing [15]. Thus, if the number of headlines in *H* is smaller than the Miller optimum, detailization is applied for the headline *T* with the largest remarkability, whereas subsumption will be applied if the number of headlines in H exceeds the Miller optimum.

## Results

**Figure 3:**
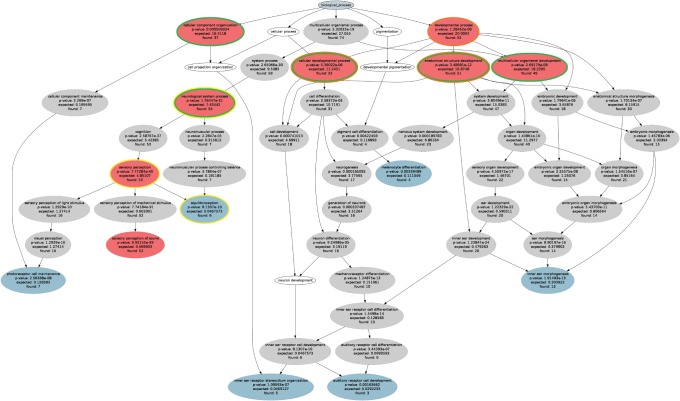
Functional abstraction of ORA results. Graphical representation of the specific ontology showing the polyhierarchy of functional annotations (GO terms) assigned to HHI gene set (G = 119, Table 1). ORA resulted in 71 terms at a significance level of p = 1.0 · 10^−2^ and Bonferroni α correction (grey ellipses). The functional abstraction approach to ORA results uses as a main measure the degree of remarkableness, calculated as the product (AND) of certainty, i.e., how safely one can assume that a GO term described the given set of genes, and information, calculated as Shannon information. Among most remarkable terms (n = 8, red ellipses), immediate redundancy is eliminated by deleting all terms that are already presented by others. This resulted in functional areas (red ellipses with green margins) conferring a comprehensive set of headline terms characterizing the biological functions of the HHI gene set. Although the present data set was of limited complexity, greater data sets may result in the initial identification of more than the desired up to nine functional areas. In this case, the method of subsumption can be applied to reduce this number. In the present case, this would, for example, join “cellular developmental process” and “anatomical structure development” to the next upper remarkable GO term “developmental process” (orange margins). In the opposite case, if the number of functional areas is low and an increase may be desirable, detailization may be applied. In this case, the terms downstream the hierarchy with the next highest remarkability are chose. For example, “neurological system process” would be split into “sensory perception and “equilibrioception” (yellow margins), which along the hierarchy have the next highest value of remarkability following the initial term. Note that the intermediate terms have lower remarkability and are therefore not chosen (Table S1).

For the HHI sample gene set (n = 119) an ORA with p-value threshold of *t_p_* = 1.0 · 10^−2^ and Bonferroni α correction resulted in the specific ontology of 71 significant terms (see Table S1) including seven details shown in figures 1 and 2. Functional abstraction identified a set *H* of *k* = 8 terms (Table 3, red in Figure 3) as headlines of the biological processes in which the 119 genes of hereditary hearing loss are involved. Subsequently, three headlines were eliminated since they were explained (covered) by other members of *H*. The final set of functional areas emerging from functional abstraction (FA) contained five terms (green circles in Figure 3). This improved the overall values of the four predefined major abstraction requirements substantially, i.e., certainty, coverage, information value and conciseness (Table 4), as compared to the currently most often used approaches to ORA interpretation (CLASSIC detail, CLASSIC p-value).

**Table 3:** The headlines produced by functional abstraction resulted in these five headline terms or functional areas (green circled red ellipses in Figure 2).

Go Term ID	GO category	Info [%]	Certainty [%]	Remarkableness	Nr. Genes (and %)
**GO:0050877**	neurological system process	97	95	92	55 (46)
**GO:0048856**	anatomical structure development	98	77	75	54 (45)
**GO:0007275**	multicellular organismal development	98	65	64	52 (44)
**GO:0048869**	cellular developmental process	98	45	44	35 (29)
**GO:0016043**	cellular component organization	100	20	20	40 (34)

Significant GO terms are a result of over-representation analysis (ORA) of the *n* = 119 genes (Table 1) of the Hereditary Hearing Impairment (HHI) gene set. The precise definition of the GO terms can be obtained using AmiGO search tool for GO at http://amigo.geneontology.org/ [20]. For a full list of significant terms and associated p-values, see Table S1. Remarkableness of a term is the product of the certainty that the term is not by chance associated with the GO biological process and the information of the particular subset of genes associated with the term. Genes is the number of genes annotated to the headline.

**Table 4:** Comparison of different abstraction methods, numerically quantified for the four required performance dimensions.

Method	Mean Certainty	Coverage	Information Value	Conciseness (ideal 5–9)
CLASSIC p-values (<10^−12^)	94%	57%	94%	8
CLASSIC details	13%	100%	50%	7
**Functional abstraction (FA)**	**61%**	**100%**	**98%**	**5**

ORA conditions of classic approaches as in Figure 1. For results of FA, see Figure 3.

## Discussion

A typical ORA results in an all-embracing, encyclopedical representation of the knowledge about biological processes, molecular functions or cellular components related with a given gene set. Human comprehension of this complex knowledge requires abstraction to a manageable number of headline terms as acknowledged previously [8]. The method of functional abstraction (FA) exceeds previous attempts of ad-hoc selections of suitable terms and uses quantifiable key requirements of an abstraction of a polyhierarchy, i.e., certainty, coverage, information value and conciseness. The method provided the comparatively highest overall values in these dimensions and identified headlines that reflect the definition of the trait exemplified by hereditary deafness [9].

The present FA method uses the term with the largest numerical value of remarkableness of each taxonomy as a candidate for a headline. The optimization of remarkableness encompasses both, the certainty that a GO term represents the taxonomy and its information value, because it is the product of both numerical values rescaled to the unit interval. By the selection of suitable terms for all taxonomies, FA also delivers the complete coverage of a selected ontology. By taking the Miller optimum into account, an abstraction of a set of headlines is obtained which explicitly aims at maximizing human understanding of the “big picture” of a specific ontology.

The process of abstraction may enable emergence [17] in the sense that novel, formerly unseen properties on a macroscopic level become visible on top of the only locally defined pieces of knowledge. Emergence in understanding might be obtained by integrating taxonomies into a more comprehensive view on the specific ontology as a whole, i.e., by the interactions of the locally defined headlines for the detail knowledge representations with the global structure of the specific ontology [17]. The procedures of detailization or subsumption provide a basis to obtain emergence in particular when in larger data sets the initial number of functional areas selected on the basis of remarkability and coverage differs from the Miller optimum [15].

In applications with larger sets of genes than in HHI this could be already observed and used for the discovery of new bits of knowledge: by a combined proteomic and transcriptomic analysis of a set of n = 231 genes were identified for the human olfactory bulb [11]. A suitable ORA identified for this gene set a set of 94 significant GO terms. By the functional abstraction method presented here the existence of neurogenesis in the adult human olfactory bulb emerged as a major finding [11]. An ORA on genes related to pain [18] resulted for the n = 410 genes causally involved in pain initially in 234 significant terms. Functional abstraction identified only 12 relevant functional areas that comprehensively describe the biology of pain from a genetics [18].

With its regard to several intuitively important dimensions of an abstraction of ORA results (certainty, coverage, information value and conciseness), FA exceeds the currently most often applied method of selecting the terms with the most significant p-values (CLASSIC), which, in contrast to FA, only aims at certainty. Such a limited focus may result in low values for coverage and information. This applied also to the present HHI example gene set. Similarly, another classical method consisting of selecting the leaves of the ontology (CLASSIC details) as an abstraction provides complete coverage. However, this method disregards information value and certainty. Moreover, the obtained headlines directly depend on the ORA parameters. In the extreme, those consist of just the root term if the chosen p-value threshold is very low, or in a great number of headlines in the opposite case. A typical example of the current state-of-the-art in the abstraction of specific ontologies is the selection of headlines for a set of 70 genes of which 55 are included in the present HHI set (Figure 4 in [8]). The specific ontology contains 49 terms and 3 details. These details and two other terms are marked as remarkable. That method of abstraction was ad hoc and involved a major subjective component.

As a consequence of this comprehensive and comprehension focused approach, FA improved the classical methods of ORA interpretation in two main ways. Firstly, it provided the number *k* of the functional areas covered by a given gene set as a result. By contrast, in the classical methods *k* depends on the selection of the p-value threshold. Secondly, FA avoided the selection of a set of terms mainly along the most important taxonomy. The reason why the CLASSIC method often results in a set covering only a single or a few but usually not all taxonomies originates from the semantics of the gene ontology. If a gene *G* is annotated to a certain term *T*, then by the rules of the GO all parents of *T* are automatically also annotated with gene G (http://www.geneontology.org/GO.annotation.conventions.shtml). Therefore, a large part of the genes annotated to term T will be also annotated to the parents (i.e. broader terms) *P* of *T*, resulting in correlated lists in *T* and *P.* If *T* is significant, the significance of P is consequently highly likely. This issue has previously been approached by a decorrelation method [5]. In their “TopGO” approach to ORA, these authors propose two different methods, ELIM and WEIGHT [5], for the recalculation of p-values based on different heuristics to eliminate correlations. Results these methods applied to the present HHI gene set are shown in the supporting information (Figures S1 and S2, respectively). The methods produced comparatively lower values in the quality dimensions of abstraction (Table 4). Moreover, GO terms emerged as significant which were not part of the original ORA results.

## Conclusions

The method of functional abstraction (FA) aims at human comprehension of voluminous gene set specific ontologies. The idea was to select terms that provide a comprehensive, yet complete coverage of the biological functions of a given gene set. The objective was achieved by (i) introducing a measure of remarkableness of a term addressing both, the certainty that a term indeed describes the functions of the gene set and the information content that avoids too general or too narrow descriptions, (ii) by selecting headlines from the most remarkable terms in order to obtain complete coverage of all parts of the polyhierarchical structure of the biological functions of the gen set, (iii) and by adjusting the number of headlines close to the Miller optimum of 5–9 to enhance human comprehension [15]. The result was an improvement of the current state-of-the art approaches to ORA interpretation in several ways. This included the identification of the number of informative headlines and the concise coverage of the original ORA. In this respect, FA exceeded the classical approaches at ORA abstraction (CLASSIC detail, CLASSIC p-value). By focusing on information rather than decorrelation of GO terms, it targeted towards human comprehension more than ELIM and WEIGHT [5] which aim at term decorrelation. On large gene sets typically obtained from topical searches or microarray analyses FA describes complex and unmanageable knowledge representations in a comprehensive manner [10], [11]. This may lead to a stimulation of the research of new aspects strengthening functional genomics in biomarker and drug discovery.

## Supporting Information

Figure S1 **ORA results and functional areas obtained with the ELIM TopGO method** [**5**] **.** Graphical representation of the specific ontology showing the polyhierarchy of functional annotations (GO terms) assigned to HHI gene set (G = 119) and forming a directed acyclic graph (DAG). The figure was generated with the GeneTrail web-based analysis tool [12]. Significant GO terms were identified using ORA, which resulted in 71 terms at a significance level of p = 1.0 · 10-2 and Bonferroni α correction (grey ellipses in which the observed number of member genes, the expected number of genes by chance and the p-value of the significance of the deviation from the expectations (Fisher’s exact test) are annotated). The TopGO approach [5] to GO abstraction exploits the correlation of terms. The selection of the k terms of the smallest values is done from the recalculated p-values. The ELIM method investigates the nodes in the GO graph bottom-up and iteratively removes genes from significant nodes [5], recalculating the ORA with the remaining set of genes. This may result in the selection of terms that were not significant in the original ORA (given at the right bottom of the figure, in red to emphasize the formal equivalence with the functional areas in Figures 1 and 3 of the main report).
(EPS)

Figure S2 **ORA results and functional areas obtained with the WEIGHT TopGO method** [**5**] **.** Graphical representation of the specific ontology showing the polyhierarchy of functional annotations (GO terms) assigned to HHI gene set (G = 119) and forming a directed acyclic graph (DAG). The figure was generated with the GeneTrail web-based analysis tool [12]. Significant GO terms were identified using ORA, which resulted in 71 terms at a significance level of p = 1.0 · 10-2 and Bonferroni α correction (grey ellipses in which the observed number of member genes, the expected number of genes by chance and the p-value of the significance of the deviation from the expectations (Fisher’s exact test) are annotated). The TopGO approach [5] to GO abstraction exploits the correlation of terms. The selection of the k terms of the smallest values is done from the recalculated p-values. In the WEIGHT method, significance scores of connected nodes (a parent and its child) are compared to detect locally most significant terms, which is achieved by down-weighting genes in less significant neighbors [5]. This may result in the selection of terms that were not significant in the original ORA (given at the right bottom of the figure, in red to emphasize the formal equivalence with the functional areas in Figures 1 and 3 of the main report).
(EPS)

Table S1 **Significant GO terms are a result of over-representation analysis (ORA) of the n = 119 genes of the Hereditary Hearing Impairment (HHI) gene set.** The precise definition of the GO terms can be obtained using AmiGO search tool for GO at http://amigo.geneontology.org/ [20]. Remarkableness of a term is the product of the certainty that the term is not by chance associated with the GO biological process and the information of the particular subset of genes associated with the term. Genes is the number of genes annotated to the headline.
(DOCX)
