# Supplementary material for: Functional Abstraction as a Method to Discover Knowledge in Gene Ontologies
Source: PLoS One. 2014 Feb 25;9(2):e90191. doi: 10.1371/journal.pone.0090191 (PMC3935416; doi:10.1371/journal.pone.0090191)
Supplement: Table S1 — Significant GO terms are a result of over-representation analysis (ORA) of the n = 119 genes of the Hereditary Hearing Impairment (HHI) gene set. The precise definition of the GO terms can be obtained using AmiGO search tool for GO at http://amigo.geneontology.org/ [20]. Remarkableness of a term is the product of the certainty that the term is not by chance associated with the GO biological process and the information of the particular subset of genes associated with the term. Genes is the number of genes annotated to the headline. (DOCX) [file pone.0090191.s003.docx]

| GoTermID | GO Category | -log10P-Value | Remarkability [%] |
| --- | --- | --- | --- |
| GO:0007605 | sensory perception of sound | 91.97 | 97.9 |
| GO:0050954 | sensory perception of mechanical stimulus | 90.02 | 96.6 |
| GO:0007600 | sensory perception | 37.93 | 94.9 |
| GO:0050890 | cognition | 35.37 | 93.6 |
| GO:0050877 | neurological system process | 29.41 | 91.8 |
| GO:0003008 | system process | 27.96 | 88.3 |
| GO:0048839 | inner ear development | 24.89 | 76.6 |
| GO:0043583 | ear development | 22.79 | 75.4 |
| GO:0007423 | sensory organ development | 18.07 | 76.1 |
| GO:0032501 | multicellular organismal process | 17.22 | 65.3 |
| GO:0042471 | ear morphogenesis | 16.2 | 59.6 |
| GO:0042472 | inner ear morphogenesis | 15.99 | 56.6 |
| GO:0060113 | inner ear receptor cell differentiation | 13.39 | 45.3 |
| GO:0042490 | mechanoreceptor differentiation | 12.49 | 44.5 |
| GO:0048856 | anatomical structure development | 11.61 | 75.4 |
| GO:0050957 | equilibrioception | 11.59 | 34.4 |
| GO:0048562 | embryonic organ morphogenesis | 11.04 | 53 |
| GO:0048731 | system development | 10.74 | 72.6 |
| GO:0048513 | organ development | 9.81 | 71.9 |
| GO:0050953 | sensory perception of light stimulus | 9.27 | 51.8 |
| GO:0007601 | visual perception | 9.27 | 51.8 |
| GO:0032502 | developmental process | 8.88 | 65.9 |
| GO:0060119 | inner ear receptor cell development | 8.83 | 27.3 |
| GO:0007275 | multicellular organismal development | 8.69 | 64.2 |
| GO:0048568 | embryonic organ development | 8.23 | 45.4 |
| GO:0050885 | neuromuscular process controlling balance | 7.87 | 30.9 |
| GO:0009887 | organ morphogenesis | 7.71 | 53 |
| GO:0045494 | photoreceptor cell maintenance | 7.29 | 26.6 |
| GO:0048598 | embryonic morphogenesis | 7.18 | 43.3 |
| GO:0009653 | anatomical structure morphogenesis | 7.1 | 53.8 |
| GO:0060122 | inner ear receptor stereocilium organization | 6.74 | 19.3 |
| GO:0043954 | cellular component maintenance | 6.35 | 23 |
| GO:0042491 | auditory receptor cell differentiation | 6.2 | 20.2 |
| GO:0050905 | neuromuscular process | 5.8 | 23.7 |
| GO:0009790 | embryonic development | 5.66 | 38 |
| GO:0048869 | cellular developmental process | 5.37 | 44.4 |
| GO:0007399 | nervous system development | 4.84 | 38.5 |
| GO:0030154 | cell differentiation | 4.74 | 38.7 |
| GO:0022008 | neurogenesis | 4.66 | 27.6 |
| GO:0030182 | neuron differentiation | 4.24 | 21.2 |
| GO:0048699 | generation of neurons | 3.65 | 16.1 |
| GO:0016043 | cellular component organization | 3.63 | 19.9 |
| GO:0048468 | cell development | 3.19 | 13.8 |
| GO:0060117 | auditory receptor cell development | 2.64 | 3.7 |
| GO:0030318 | melanocyte differentiation | 2.28 | 2.5 |
| GO:0050931 | pigment cell differentiation | 2.19 | 2.1 |
| GO:0005201 | extracellular matrix structural constituent | 7.07 | 30.9 |
| GO:0003779 | actin binding | 4.69 | 26.5 |
| GO:0005516 | calmodulin binding | 4.69 | 21.1 |
| GO:0003774 | motor activity | 4.66 | 18.9 |
| GO:0008092 | cytoskeletal protein binding | 3.86 | 17.6 |
| GO:0030020 | extracellular matrix structural constituent conferring tensile strength | 4.67 | 11.2 |
| GO:0005243 | gap junction channel activity | 3.16 | 5 |
| GO:0000146 | microfilament motor activity | 2.61 | 4.1 |
| GO:0022829 | wide pore channel activity | 2.38 | 3.7 |
| GO:0032420 | stereocilium | 19.88 | 56.2 |
| GO:0032421 | stereocilium bundle | 18.97 | 55.3 |
| GO:0005902 | microvillus | 14.83 | 53.5 |
| GO:0042995 | cell projection | 4.91 | 36.6 |
| GO:0005581 | collagen | 7.41 | 29.6 |
| GO:0016459 | myosin complex | 6.63 | 27.6 |
| GO:0015629 | actin cytoskeleton | 4.6 | 21 |
| GO:0005578 | proteinaceous extracellular matrix | 4.05 | 17.5 |
| GO:0031012 | extracellular matrix | 4.04 | 17.3 |
| GO:0044420 | extracellular matrix part | 4.44 | 15.6 |
| GO:0031941 | filamentous actin | 4.77 | 15 |
| GO:0005921 | gap junction | 4.55 | 12.6 |
| GO:0005922 | connexon complex | 3.78 | 8.2 |
| GO:0005884 | actin filament | 3.63 | 7.6 |
| GO:0005911 | cell-cell junction | 2.31 | 5 |
| GO:0045177 | apical part of cell | 2.17 | 2.8 |
| GO:0005587 | collagen type IV | 2.34 | 2.7 |
| GO:0016461 | unconventional myosin complex | 2.1 | 1 |
| GO:0030935 | sheet-forming collagen | 2.1 | 1 |
| GO:0016324 | apical plasma membrane | 2.01 | 0.7 |
